# Clinical Significance and Role of TK1, CEA, CA 19-9 and CA 72-4 levels in Diagnosis of Colorectal Cancers

**DOI:** 10.31557/APJCP.2020.21.11.3133

**Published:** 2020-11

**Authors:** Swarnima Singh, Rakesh Kumar, Uday Kumar, Rekha Kumari

**Affiliations:** 1 *Department of Biochemistry, Netaji Subhas Medical College and Hospital, Patna, India.*; 2 *Department of Surgical Gastroenterology and Liver Transplant, Indira Gandhi Institute of Medical Sciences, Patna, India. *; 3 *Department of Biochemistry, Indira Gandhi Institute of Medical Sciences, Patna, India. *

**Keywords:** Thymidine kinase 1, colorectal cancer, tumor marker, CEA

## Abstract

To explore the diagnostic value, pre-operative serum thymidine kinase 1(TK1), CEA, CA 19-9 and CA 72-4 levels were measured in 106 patients with colorectal carcinoma (53 colon and 53 rectal carcinoma patients) and 53 healthy controls. Sandwich Elisa, biotin-labeled antibody kit was used for TK1, and other tumor markers were measured using electro-chemiluminescence. Serum TK1 levels were significantly higher in CRC than in healthy controls (p<0.05) and showed significant associations with tumor stage, histopathological grade, lymph node status and metastasis (p<0.01). TK1 showed the highest (0.824-0.862) area under receiver operating characteristics curve (AUC) in comparison to other markers, and the AUC of the panel of combination tests performed even better (0.935-0.952). Significant variation was observed between the single biomarker test and their combination (Z test, p<0.01) and the Hosmer-Lemeshow test showed an adequate model of calibration. The algorithm based on combination of TK1, CEA, CA19-9 and CA72-4 can improve the diagnostic efficiency in CRC patients.

## Introduction

Colorectal cancer (CRC) is a formidable health problem worldwide and the third common explanation for cancer death (GLOBOCAN, 2018 ). In India, the five year survival of CRC is lowest in the world at less than 40% (Allemani et al., 2015). The importance of early diagnosis stems from the fact that the five year survival rate is around 90% for localized tumor, 70% for regional and only 13% for metastatic CRC (Siegel et al., 2016).

In clinical practice, endoscopic procedures (colonoscopy, sigmoidoscopy) and computed tomography (CT) colonography are used to diagnose CRC. Due to their complex techniques, high costs, requirement of repeating (3-5 years), they have poor compliance rates (Garborg et al., 2013; Anderson et al., 2011) especially in developing countries and rural areas.

The blood-based tumor markers are being emphasized as simple, relatively inexpensive, reproducible, quantitative, and objective diagnostic tool. One such glycoprotein carcinoembryonic antigen (CEA) is widely used CRC molecular marker, but its association with other types of cancer (ovarian cancer, inflammatory bowel disease) has jeopardized its specificity (Yanfeng et al., 2018). Other serum markers like cancer antigen 19-9 (CA19-9) (Wang et al., 2002), cancer antigen 72-4 (CA72-4) (Carpelan-Holmstrom et al., 2004), have also been utilized for CRC diagnosis, post-operative surveillance, and treatment monitoring, but inconsistent results have been obtained.

Thymidine kinase (TK) catalyzes transformation of deoxythymidine to thymidine monophosphate (TMP) in the presence of adenosine triphosphate (ATP), and is an important marker of cell proliferation. TK has two isoenzymes- TK1 found in cytoplasm is cell-cycle dependent and TK2 in mitochondria is cell-cycle independent. TK1 concentration and activity are augmented at the late G1 stage of the cell cycle, reaches its peak at the late S-phase/early G2 stages, and the levels tend to downgrade at the mitotic stage. TK1 is a salvage enzyme and degrades intracellularly. But in tumor cells, due to rapid cell division, it spills into circulation and serves as a tumor marker. TK1 has high sensitivity to be used as primary diagnostic tool (Svobodova et al., 2007). Studies have well correlated TK1 with disease severity and as predictive tumor marker for monitoring the disease course (Du Ying-Ying et al., 2016 ). 

Due to the highly heterogenous nature of CRC, with a vast array of mutations and mutagens, a panel of tumor markers can be a more viable approach to diagnosis. In this study we explored the diagnostic value of joint detection of Thymidine Kinase 1(TK1), carcinoembryonic antigen (CEA), carbohydrate antigen 19-9 (CA 19-9) and carbohydrate antigen 72-4 (CA 72-4) in the diagnosis of CRC, and evaluated the relationship between TK1 expression and clinical pathological characteristics in the patients.

## Materials and Methods

Participants: A sample size of 106 CRC patients was calculated, guided by previous literature (Ning et al., 2018), considering TK1, CEA as primary outcome variables, with 5% level of significance and 95% CI (confidence interval). Further to compare TK1 levels with healthy controls, 53 controls were selected after Institutional ethical clearance (431/IEC/2018/IGIMS). The inclusion criteria were: histologically confirmed case of CRC with informed consent and agreement for additional markers. Exclusion criteria were: previously treated case of CRC, pregnancy, significant concomitant diseases such as chronic heart failure, and severe chronic liver or renal disease.

Laboratory analysis: Pre-operative blood samples were collected in serum separator tube (SST) and allowed to clot for 30 minutes at room temperature before centrifugation for 15 minutes at 1,000xg. Serum was removed, aliquoted and stored at <20^o^C, for analysis within 3 months. Serum TK1 concentrations were measured using solid phase quantitative sandwich enzyme immunoassay technique (LSBio), according to manufacturer’s protocol. Normal reference for TK1 was assumed to be 0-2.0 pmol/L (Ning et al., 2018; https://www.lsbio.com/elisakits/human-tk1-tk-thymidine-kinase-sandwich-elisa-elisa-kit-ls-f11880/11880). Serum concentrations for CEA, CA19-9 and CA72-4 were measured by electrochemiluminescence (Cobas, Roche Diagnostics) and normal reference range were 0-6.5 ng/ml, 0-37 U/ml and 0-5.7U/ml, respectively.

*Statistical analysis*

Data was analyzed on SPSS 20.0 software. Correlation between serum TK1 levels and clinicopathalogical parameters were checked by chi square test. Logistic regression and Receiver operating characteristic (ROC) were analyzed, area under the ROC curve (AUC) were drawn, 95% CI and Youden’s index were calculated for each tumor marker and their combination, and Hosmer-Lemeshow test was used for assessment. Sensitivity and specificity were computed using Youden’s index. AUCs of individual markers and combination test was compared by Z test. The level of significance was set at p<0.05.

## Results

All 106 patients had tumor histology revealing adenocarcinoma and commonest primary site was rectum/anorectum (53,50%), followed by recto-sigmoid (21,20%), and colon (32,30%). With a mean age of 49.2 years, 68% patients were male. Most CRC patients were in the age group 40-60 years, while 30% were below 40 years of age. Most patients were in stage III (53,50%), while 30 (28.3%) had stage IV, 18 (17%) stage II and only 5 (4.7%) in stage I disease, according to the AJCC (American Joint Committee on Cancer Staging), Tumor, Lymph nodes, Metastasis (TNM) staging classification. TK1 concentrations were significantly higher in CRC patients that in healthy controls (p<0.05). TK1 levels showed a positive correlation with tumor stage, grade, lymph node involvement and presence of metastasis (table I). From diagnostic point of view, the use of TK1 had good specificity (97.2-97.4%), but poor sensitivity (58-66.8%) for colon and rectal cancers (table II). Nevertheless, diagnostic sensitivity improved (80.9-86.9%), without compromising the specificity, when all the four markers were used in combination. When the tumor markers (TK1, CEA, CA 19-9 and CA 72-4) were detected respectively, the AUC of TK1 for the colon cancer was highest (0.862), and second highest in rectal cancer (0.824). However, the combination of AUC was higher than that for each tumor marker detected respectively (0.935-0.952), and the Hosmer-Lemeshow test showed an adequate model of calibration. Moreover, the AUCs varied significantly between the combination tests and single biomarker tests (Z test, P<0.01). 

**Table 1:** Association between TK1 Levels and Clinicopathological Characteristics of CRC Patients

Clinicopathological feature	Number of patients	TK1<= 2.0 pmol/L	TK1>2.0 pmol/L	p value
T stage				<0.001
T1+T2	18	13	5	
T3+T4	88	20	68	
Lymph node status				0.016
Negative	20	11	9	
Positive	86	22	64	
Metastasis				<0.001
Absent	76	32	44	
Present	30	1	29	
Histological differentiation				0.003
Well + Moderate	51	23	28	
Poor + Undifferentiated	55	10	45	
Stage				0.005
I + II	23	13	10	
III + IV	83	20	63	

**Table 2:** Area Under the Receiver Operating Curve (AUC) for TK1, CEA, CA19-9 and CA72-4 for Colorectal Cancer Patients (colon cancer, 53; rectal cancer, 53) Versus Healthy Controls

Tumor markers	Sensitivity (%)	Specificity (%)	AUC	p-value
Colon cancer (N=53)				
TK1	66.8	97.2	0.862	<0.001
CEA	64.9	90.2	0.791	<0.001
CA19-9	62.0	89.2	0.736	<0.001
CA72-4	45.3	95.9	0.745	<0.001
Combination	86.9	96.0	0.952	<0.001
Rectal cancer (N=53)				
TK1	58.0	97.4	0.824	<0.001
CEA	56.4	97.4	0.827	<0.001
CA19-9	48.4	93.2	0.683	<0.001
CA72-4	69.4	63.9	0.707	<0.001
Combination	80.9	96.1	0.935	<0.001

## Discussion

We could not find literature evaluating serum TK1 alone or in combination in Indian population with CRC. Weagel EG et al found significant surface localization of TK1 in CRC patients by flow cytometry and confocal microscopy and recommended it as a potential clinical biomarker (Weagel et al., 2018). Herein significantly raised serum TK1 levels were noticed in CRC group than in healthy controls. Similar findings were reported by Bolayirli et al., (2013) and Ning et al., (2018). A meta-analysis revealed that serum TK1 significantly distinguished healthy persons and those with benign colorectal tumors from patients with CRC (p<0.000001). It also highlighted that benign tumor patients with serum TK1 values > 2.0 pmol/L had significantly higher chances of developing malignant colorectal tumors (Dang et al., 2020).

Serum TK1, in our study, showed significant correlation with tumor’s clinicopathological stage and differentiation. Ning et al found significant correlation between serum TK1 with stage, lymph node involvement, metastasis, tumor size, and age among 344 colorectal and 169 gastric carcinoma participants (Ning et al., 2018). Liu et al., (2011) (in their study on gastric carcinoma patients, reported rise in mean TK1 values from stage I+II to stage III+IV. A meta-analysis suggested significantly worse survival among CRC patients with elevated TK1 levels (>0.9 pmol/L) in comparison to those with low TK1 values (<=0.9 pmol/L) and cox regression analysis showcased TK1, clinicopathological stage and lymph node status to be independent factors for prognosis. TK1, as a biomarker, could be effective for early detection of benign colorectal lesions to halt their progress into malignancy (Dang et al., 2020).

We reported the sensitivity of CEA in the range of 56.4-64.9%, which is consistent with previous observations (Yanfeng et al., 2018; Ning et al., 2018; Fakih and Padmanabhan, 2006) Although there is no significant difference on the highest sensitivities among CEA and TK1, the combination of TK1+ CEA+ CA 19-9 + CA 72-4 was able to attain markedly improved diagnostic sensitivity (80.9-86.9%). Yanfeng et al., (2018) advocated the use of CEA+ CA 19-9 + CA72-4 + CA125 with a diagnostic sensitivity of 66.7% for accurate and cost-effective results. Another study offered a combination of TK1+ CEA+ CA19-9 + CA72-4 for diagnostic sensitivity of 80.8-87% for CRC and 88.2% for gastric carcinoma (Ning et al., 2018).

The AUC’s for CA19-9 and CA 72-4 ranged from 0.683-0.745, indicating that both these markers were lacking in accuracy that was close to random for CRC diagnosis. With an AUC score between 0.824-0.826, TK1 looked quite specific and could be projected as an independent marker for CRC diagnosis. Nevertheless, the joint detection of TK1+CEA + CA19-9 + CA72-4 offered a better diagnostic performance in the logistic regression model, which is consistent with some previous findings also (Liang et al., 2016; Ning et al., 2018; Yanfeng et al., 2018, Dang et al., 2020).

Limitations: These are hospital referred patients and do not necessarily reflect the true distribution of CRC subsites. Since the organ specificity of TK1 is low (Svobodova et al., 2007), effect of other cancers and non-specific causes (inflammatory or immunological) can also be studied.

Conclusions : Serum TK1 may be a self-reliant diagnostic tool for CRC patients (based on AUC score), and the combination of TK1, CEA, CA 19-9 and CA 72-4 scored even better. We deduced that joint evaluation of four tumor markers may be substantial for early diagnosis of CRC. TK1 levels correlates well with disease severity. It may have a role in prediction of disease relapse, and serve as immunotargets for monoclonal antibodies.

## References

[B1] Allemani C, Weir HK, Carreira H (2015). Global surveillance of cancer survival 1995–2009: analysis of individual data for 25,676,887 patients from 279 population-based registries in 67 countries (CONCORD-2). Lancet.

[B2] Anderson JC, Fortinsky RH, Kleppinger A (2011). Predictors of compliance with free endoscopic colorectal cancer screening in uninsured adults. J Gen Intern Med.

[B3] Bolayirli M, Papila C, Korkmaz GG (2013). Serum thymidine kinase l activity in solid tumor (breast and colorectal cancer) patients treated with adjuvant chemotherapy. J Clin Lab Anal.

[B4] Carpelan-Holmström M, Louhimo J, Stenman U (2004). CEA, CA 242, CA 19-9, CA 72-4 and hCG beta in the diagnosis of recurrent colorectal cancer (2004). Tumor Biol.

[B5] Dang L, Ma H, Hei A (2020). A meta analysis of serological thymidine kinase 1 as a marker for colorectal benign and malignant tumor risk assessment. Mol Clin Oncol.

[B6] Du Y-Y, Zhang Q-J, Sun G-P (2016). Expression and clinical significance of cytokeratin-19 and thymidine kinase -1 in advanced gastrointestinal cancer. China Med J.

[B7] Fakih MG, Padmanabhan A (2006). CEA monitoring in colorectal cancer. What you should know. Oncology (Williston Park).

[B8] Feng F, Tian Y, Xu G (2017). Diagnostic and prognostic value of CEA, CA19–9, AFP and CA125 for earlygastriccancer. BMC Cancer.

[B9] Garborg K, Holme Ø, Løberg M (2013). Current status of screening for colorectal cancer. Ann Oncol.

[B11] LiangY, Wang W, Fang C (2016). Clinical significance and diagnostic value of serum CEA, CAl9-9 and CA72-4 in patients with gastric cancer. Oncotarget.

[B12] Liu Y, Ling Y, Qi Q (2011). Changes in serum thymidine kinase 1 levels during chemotherapy correlate with objective response in patients with advanced gastric cancer. Exp Ther Med.

[B13] Ning S, Wei W, Li J (2018). Clinical significance and diagnostic capacity of serum TK1, CEA, CA 19-9 and CA 72-4 levels in gastric and colorectal cancer patients. J Cancer.

[B14] Siegel RL, Miller KD, Jemal A (2016). Cancer statistics, 2016. CA Cancer J Clin.

[B15] Svobodova S, Topolcan O, Holubec L (2007). Prognostic importance of thymidine kinase in colorectal and breast cancer. Anticancer Res.

[B16] Wang WS, Lin JK, Chiou TJ (2002). CA19-9 as the most significant prognostic indicator of metastatic colorectal cancer. Hepatogastroenterology.

[B17] Weagel EG, Burrup W, Kovtun R (2018). Membrane expression of thymidine kinase 1 and potential clinical relevance in lung, breast, and colorectal malignancies. Cancer Cell Int.

